# Developments in pediatrics in 2020: choices in allergy, autoinflammatory disorders, critical care, endocrinology, genetics, infectious diseases, microbiota, neonatology, neurology, nutrition, ortopedics, respiratory tract illnesses and rheumatology

**DOI:** 10.1186/s13052-021-01184-4

**Published:** 2021-12-07

**Authors:** Carlo Caffarelli, Francesca Santamaria, Michela Procaccianti, Ettore Piro, Valeria delle Cave, Melissa Borrelli, Angelica Santoro, Federica Grassi, Sergio Bernasconi, Giovanni Corsello

**Affiliations:** 1 Clinica Pediatrica, Department of Medicine and Surgery, Azienda Ospedaliera-Universitaria, University of Parma, Via Gramsci, 14 Parma, Italy; 2 Department of Translational Medical Sciences, Federico II University, Naples, Italy; 3 Department of Sciences for Health Promotion and Mother and Child Care ‘’G. D’Alessandro”, University of Palermo, Palermo, Italy; 4 Microbiome Research Hub, University of Parma, Parma, Italy

**Keywords:** Allergy, autoinflammatory disorders, critical care, endocrinology, genetics, infectious diseases, microbiota, neonatology, neurology, nutrition, ortopedics, respiratory tract illnesses, rheumatology

## Abstract

In this article, we describe the advances in the field of pediatrics that have been published in the Italian Journal of Pediatrics in 2020. We report progresses in understanding allergy, autoinflammatory disorders, critical care, endocrinology, genetics, infectious diseases, microbiota, neonatology, neurology, nutrition, orthopedics, respiratory tract illnesses, rheumatology in childhood.

## Introduction

This review focuses on a critical evaluation of latest advances in epidemiology, etiopathogenesis and clinical features of selected areas of pediatrics. Reports determining progresses in allergy, autoinflammatory disorders, critical care, endocrinology, genetics, infectious diseases, microbiota, neonatology, neurology, nutrition, orthopedics, respiratory tract illnesses, rheumatology were identified and chosen through the eyes of most accessed papers that were published in the Italian Journal of Pediatrics (IJP) in 2020. These papers offer remarkable data to improve care of children with problematic disorders.

### Allergy. 1- COVID-19; 2- Atopic dermatitis; 3- Food protein induced enterocolitis syndrome

#### COVID-19

The first cases of the coronavirus disease (COVID-19) caused by severe acute respiratory syndrome coronavirus-2 (SARS-CoV-2) were identified in Wuhan, China on 31 December 2019. Since then, huge efforts have been made to understand how to manage children with chronic disease including allergy or immunological diseases during the COVID-19 pandemic. The earliest Italian epidemiological data [1] showed that 20% of children with COVID-19 were affected by allergic rhinoconjunctivitis and 10-20% by asthma. Moreover, asthma or allergic rhino-conjunctivitis did not represent a risk factor for more severe COVID-19. At the onset of the disease, there were large lack of information about understanding how to manage children with chronic diseases including allergy or immunological diseases during the COVID-19 pandemic. A major highlight of IJP was the publication of the expert panel consensus document from the Italian Society of Pediatric Allergy and Immunology (SIAIP) [2] that helped decision-making with respect to visits, diagnostic tests and treating children and adolescents with allergic rhinitis, asthma, atopic dermatitis, allergy to drugs, food, hymenoptera venom or latex, and primary immunodeficiencies during COVID-19 pandemics. Preventing measures aimed at reducing the risk of infection for health care provider and patients were described. They mainly consisted of personal protective equipment, sanitization of medical tools and minimum safe distance. In the upper airways, the pathological changes due to infection and allergic rhinitis [3] frequently coexist, therefore a multidisciplinary clinical approach should be considered [4]. In pandemic time, respiratory function tests in asthmatics should be performed only if urgent when patients and caregivers do not present flu-like symptoms [2]. Unnecessary exhaled nitric oxide measurement [5, 6] should be avoided and research studies on exhaled biomarkers [7] carefully conducted. Disposable filter, instrument sterilization, and personal protective equipment should be used during visits or other diagnostic procedures such as skin test to allergens [8] or blood sampling for IgE detection to inhalants [9] and foods [10] and for autologous serum skin test [11]. Anaphylaxis, food protein-induced enterocolitis syndrome, allergy to nutritionally essential foods, severe reactions to hymenoptera venoms, reactions to necessary drugs, severe eczema or asthma need a rapid allergy workup. Oral food challenges for assessing tolerance achievement [12] or when mild subjective symptoms of uncertain origin are reported [13], should be postponed. Food [14] as well as drug challenge [15] can be necessary when a safe alternative food [16] or drug [17] is unavailable. Meter dose inhalers should be preferred to nebulizers also in preschool children with wheezing irrespective of pandemics [18]. Routine vaccination for infectious diseases should be continued but patients with reactions to vaccine should be carefully managed [19] and subcutaneous immunotherapy [20] should be carried on during the COVID-19 pandemic. The task force provided “red flags” for an urgent hospital-based consultation. In other cases, mobile health technologies could have a role in improving care around immunodeficiency or allergic disease management. Telehealth medicine permitted performing consultations in children with allergy and maintaining social distancing. The MASK study showed the utility of a mobile app to improve adherence to treatment for allergic rhinitis [21]. A new mobile diary app with the platform “AllergyMonitor” simultaneously provided pollen concentrations. Digital technology was helpful for virtual assessment of children with allergic rhinitis due to pollen allergy by improving symptom prediction, selection of allergen immunotherapy composition aeroallergens [22], and adherence to treatment [23].

### Atopic dermatitis

Atopic dermatitis is a common inflammatory chronic skin disorder that mainly results in bothersome itching leading to sleep disturbance. It is often associated with food allergy [24]. The control of pruritus is often difficult to reach by pharmacological treatments, elimination diet and educational interventions. El Hachem M et al [25] highlighted the key role played by the psychological approach. It included cognitive-behavioural techniques, targeted at moving attention from itching and medication towards activities that strongly engage the patient. Distraction techniques comprised involvement, breathing, relaxation, hypnotic procedures such as visualization and desensitization. The choice depended on age, preferences, and attitudes. Distraction techniques were helpful not only for patients but also for parents and siblings.

### Food protein induced enterocolitis syndrome

Food protein induced enterocolitis syndrome is an uncommon illness due to cow’s milk and solid foods [26, 27]. In breast-fed infants, it rarely occurs, and it can be misdiagnosed on the basis of history and symptoms. The interesting report by Baldo et al [28] showed that the diagnostic workup for identifying exclusively breastfed infants with food protein induced enterocolitis syndrome was made simpler by detection of increased circulant methemoglobin. In the drive to improve identification of infants with food intolerance, this biomarker should be always determined.

### Autoinflammatory disorders. 1- Familial Mediterranean Fever

Autoinflammatory disorders are diseases primarily due to innate immunity hyperactivation in the absence of autoantibodies. Main advances on the crucial problem of Familial Mediterranean Fever (FMF) were reported by Maggio et al [29]. They recognized that genetic factors, including mutations of MEFV gene in the chromosome 16p13, encoding pyrin, mainly expressed by innate lines in patients with FMF [30] lead to inflammasome overproduction of IL-1. They pointed out that Tel Hashomer Hospital criteria [31] or the Turkish FMF Paediatric criteria [32] should be used for the diagnosis. Finally, they indicated that prevention of recurrent attacks and of long-term complications, as amyloidosis was achieved with colchicine and when it was not effective, with Canakinumab, a monoclonal antibody anti-IL-1 beta.

### Critical care- 1- Echocardiography

Hemodynamic monitoring is essential for the diagnosis and treatment of critically ill patients. Initially, physical examinations, vital signs, urine output, central venous pressure, and transthoracic echocardiography are used to evaluate the preload and afterload status and cardiac functions in response to fluid resuscitation [33]. However, as most of the above reported parameters are subjective, more advanced hemodynamic monitoring of patients in the pediatric Intensive Care Unit (ICU) is warranted. In ICU, transthoracic doppler echocardiography, pulmonary artery catheterization and transpulmonary thermodilution methods are used to measure the Cardiac Output (CO). Insertion of a pulmonary artery catheter into the right heart is invasive and can be responsible for cardiopulmonary complications [34]. Pulse index Contour Cardiac Output (PiCCO) monitor is less invasive for checking continuous CO and hemodynamics. It is based on transpulmonary thermodilution technology, does not require pulmonary artery catheterization, and only needs a central venous and a femoral artery catheter. The procedure is indicated in hemodynamically unstable patients, who also show uncertain volume status. It drives ICU specialists in planning fluid and inotrope treatment to be applied by providing information on the patient preload and systemic vascular resistance [35]. Aslan et al [36] showed a significant, positive correlation between CO and Cardiac Index levels measured by critical care echocardiography and PiCCO. Authors suggested to use echocardiography being less invasive than PiCCO, for monitoring critically ill pediatric patients.

### Endocrinology. 1- COVID-19; 2- Resistance to thyroid hormone syndrome; 3- diabetic ketoacidosis

#### COVID-19

After the long lockdown period due to the first wave of the COVID-19 pandemic in Italy, Stagi et al [37] described a significant increase, compared to previous years, in endocrinological consultations for precocious puberty and for acceleration of puberty in patients already diagnosed, particularly in females. These findings were later confirmed by a subsequent report from another pediatric hospital [38]. The causes of this phenomenon are not exactly known even if changes in lifestyles [39], decrease in physical activity, worsening of nutritional quality, increased levels of stress in the family, alteration of sleep rhythms, overuse of electronic devices may have played a role. It is however conceivable, also in the light of recent experimental models [40], that excess energy may be the fundamental element in the induction of puberty. Further studies on larger samples, especially regarding the male sex, are warranted to better understand the fascinating series of neuroendocrine and metabolic mechanisms that regulate puberty.

### Resistance to thyroid hormone syndrome

Sun et al [41] provided an up-to-date review on the clinical consequences of the alteration of the thyroid hormone receptor beta gene. This pathological situation is considered the most frequent of the forms of resistance to thyroid hormone (RTH). It has an occurrence of one case per 40 000 live births and more than 3000 cases have been published [42]. Patients are mostly asymptomatic, but occasionally present clinical characteristics of thyrotoxicosis and/or hypothyroidism. The most common biochemical features are high levels of circulating free T4 and T3, and non-suppressed serum TSH levels responding to thyrotropin-releasing hormone (TRH) test [43]. The familial occurrence of RTH has been documented in approximately 75% of cases and the inheritance is primarily autosomal dominant. For the diagnosis it is necessary to perform a molecular analysis. Based on some experiences among the forms without alteration of the beta receptor gene, those with albumin gene (ALB) mutation are most frequently found [44].

### Diabetic ketoacidosis

Ketoacidosis (DKA) in patients affected by insulin dependent type 1 diabetes (T1D) is characterized by hyperglycemia, ketosis, and metabolic acidosis (according to ISPAD- International Society for Pediatric and Adolescent Diabetes: mild form: pH < 7.30 or serum bicarbonate < 15 mmol/L) and it is considered the most important cause of death in T1D children and adolescents. Too often, DKA manifests itself at the onset of the disease. A recent retrospective survey showed that in the 2006-2016 period the incidence of DKA was 29.9% at onset of diabetes in 59,000 children diagnosed in thirteen countries [45]. Moreover, it has also been reported that DKA at diagnosis is less frequent when the disease is more common and better known. On the other hand, it has been estimated that the overall incidence of DKA in patients with established T1D varies from 1 to 12/100 person-years [46]. Particularly in the recurrent forms it is important to identify facilitating factors to adopt preventive measures. The interesting work by Assefa et al [3] performed in northern Ethiopia showed that in that geographic area the most important predictors to focus on to prevent DKA are: age <5 years, non-adherence, inappropriate insulin storage, presence of upper respiratory tract infections during diabetic ketoacidosis diagnosis and preceding gastroenteritis [47]. These are factors in line with international experiences that indicate young age, socio-economic conditions, and health care organizations as the sectors in which educational, economic aid and health aid programs are most likely to be helpful [48].

### Genetics. 1- Network of rare diseases; 2- Polycystic kidney disease; 3- CHARGE Syndrome

#### Network of rare diseases

Rare genetic diseases are estimated to be over 8000. Most of them are due to altered functions of single genes, whereas only a small proportion of rare diseases recognizes non-genetic causative factors. Appropriate and timely diagnosis improves patient health status, reducing psychological and social burden of the diseases and allowing proper genetic counselling [49]. The Italian Undiagnosed Rare Diseases Network [50] collected information of 110 cases between March 2016 and June 2019. The goal of the network was to make timely and appropriate diagnoses of the most complex disorders. The age of onset of the disease ranged from prenatal age to 51 years. Conditions were predominantly sporadic. Almost all patients had multiple organs involved; the nervous system was, indeed, the most affected together with joint/skeletal and ocular systems. Four out of 71 had liver involvement alone and in 2 additional patients systemic autoimmune and endocrine manifestations were detected, respectively. A total of 13/71 family cases were investigated by Whole Exome Sequencing. In some families more than one individual was affected, leading to 20/71 individuals investigated. Disease causing variants were identified in two cases demonstrating homozygous and heterozygous variants associated with previously undescribed phenotypes. In 5 cases new candidate genes were identified, although confirmatory tests are still pending. In 3 families, investigations were not completed due to the scarce compliance of members and molecular investigations were suspended. Finally, 3 cases (one familial) remain still unsolved. Twelve undiagnosed clinical cases were then selected to be shared at international level through PhenomeCentral. Phenome Central is a registered-access network for clinicians, researchers, and scientific consortia aimed to display and match patient’s phenotype and genotype data and discover similar patients across the world. Phenome Central simultaneously identifies patients who might have the same disease and predicts genes potentially causative.

### Polycystic kidney disease

The spectrum of genetically inherited polycystic kidney diseases (PKDs) includes the autosomal dominant polycystic kidney disease (ADPKD, which is found mostly in adults), and the autosomal recessive polycystic kidney disease (ARPKD), a severe form which is primarily reported in the perinatal period or early childhood (1:20.000 live births). The disorder is caused by mutations in the polycystic kidney and hepatic disease 1 (*PKHD1*) gene, which encodes fibrocystin (also known as polyductin), a protein localized in the primary cilium and basal body [51]. Manifestations of ARPKD include hepatic fibrosis and greatly enlarged kidneys, with cysts typically affecting the collecting ducts. Causes of early death are sepsis or respiratory failure, due to lung hypoplasia and displacement of the diaphragm by bilateral nephromegaly [52]. Mechanical ventilation usually is applied in approximately 41% of cases [53]. Serra et al [54] described a preterm female with perinatal, rapid, and bilateral, abnormal growth of both kidneys, respiratory failure, and initial signs of liver disease. Postnatally, she suffered from severe respiratory distress syndrome due to pulmonary hypoplasia, a rapid and progressive enlargement of both kidneys, severe hypertension, increased size of the liver, as well as peripheral biliary dilatation. The patient died on the 78th day of life, for a fungal sepsis which worsened respiratory insufficiency. She was affected by a rare and a severe homozygous mutation in the PKHD1 gene with functional impairment of the encoded protein (polyductin/fibrocystin, 4074 amino acids) [55]. This paper showed that next generation sequencing (NGS) techniques are becoming increasingly important as they provide accurate genomic information, avoid more invasive investigations such as renal biopsy, and allow to define the outcome of the disease, thus supporting clinicians in the choice of the most appropriate management.

#### CHARGE Syndrome

CHARGE syndrome [56–58] is characterized by a considerable phenotypic variability. Ear abnormalities are frequent (> 90%) and are an important clinical clue for diagnosis in those cases with no additional major criteria [59]. Bedeschi MF et al [59] reported on eight patients with CHARGE syndrome and described external ear shape and position anomalies, middle ear ossicular malformations with chronic otitis media, and several inner ear anomalies cochlear and vestibular anomalies, consisting of varying degrees of cochlea hypoplasia and malformations and dysplasia of semi-circular canals. The authors outlined as middle and inner ear defects can be easier found by CT scan and MRI of the temporal bone, thus allowing a prompt clinical diagnosis.

### Infectious diseases. 1- COVID-19; 2- Septic shock; 3- Dacriocystitis; 4- Thrombophlebitis

#### COVID-19

A position paper of the Italian Society of Pediatric Infectious Diseases on treatments for pediatric COVID-19 [60] after reviewing available literature as 16 June 2020, was published in IJP. This paper was extremely useful since at the time of publication only a few national societies provided recommendations on this issue [61–67]. Since most SARS-CoV-2 infections in children were asymptomatic or mild [68, 69], the report documented that supportive therapy (e.g., antipyretic therapy, parenteral rehydration, oxygen therapy) was sufficient in most cases. Pharmacological treatment was proposed to be added to supportive care in patients with severe or critical disease. The expert panel suggested to use remdesivir and if not available, hydroxychloroquine or lopinavir/ritonavir and to consider the use of methylprednisolone or interleukin inhibitors if available (Anakinra or Tocilizumab). The panel opinion mirrored the knowledge available at that time. Nowadays, it is advisable to prescribe antivirals according to the results of clinical trials that are continuously published on the effectiveness of drugs against COVID-19 or as part of a clinical trial. It is of note that the document suggested to give antibiotics only for suspicion of bacterial infection and in the presence of predisposing comorbidities (such as immunodeficiency, cystic fibrosis, other chronic diseases of the respiratory tract, severe neuromotor disability). Since in children the incidence of thrombotic complication was low, the panel did not routinely recommend preventive anticoagulant therapy, but suggested to consider it in children and adolescents with a higher risk of thrombosis, particularly when severe inflammatory conditions coexist.

Several studies have assessed the chain of SARS-CoV-2 transmission to adopt preventive measures. Facial mask has been demonstrated to be effective in preventing the spread of respiratory viruses (such as COVID-19) [70] without any harmful effects on gas exchange neither in children nor in adults even when doing mild exercise [71]. The use of the mask is recommended by the WHO and the Centers for Disease Control and Prevention (CDC) in adults [72, 73]. Several scientific societies felt the need to produce recommendations to increase awareness on the use of mask in children. However, the Japan Pediatric Society, CDC [74], the American Academy of Pediatrics [75] and the European Academy of Pediatrics [76] agreed that children under 2 years old should not wear a mask because of asphyxia risk. IJP published a position statement of the Italian Pediatric Society challenges misconception on face mask in children and disseminate scientific trustable information on its benefit [77]. In Italy, face mask is advised in all healthy children aged more than 3 years old and is mandatory over 6 years of age. This document was helpful for medical doctors and National Health Authorities that had to establish preventive measures in the general population.

The restrictive measures imposed by governments to contain the COVID-19 spread caused a sudden change in the habits and lifestyles of the population, especially regarding social distancing, eating habits, sleeping habits and everyday behaviours (digital-education, smart working, limitation of outdoor and indoor physical activity) [78, 79]. Moreover, there is a long-standing debate on school closure during the pandemics. Even if most countries closed schools, a systematic review [80] showed that school closures did not contribute to SARS-CoV-2 transmission control. Two studies conducted respectively in Australia and Ireland showed that the spread of COVID-19 within schools has been very limited [81]. The connection between the re-opening of schools in September 2020 and the spreading of infections may be mainly attributed to the public transport used by students [82]. This may be explained by the fact that during the COVID-19 pandemic up until now, children accounted for lower proportion of cases than expected from their population. Moreover, they were mostly asymptomatic or with mild disease [83]. The economic costs and secondary effect of school closure underlined that are well known [80]. Along this line, it is noteworthy the paper by Fantini et al [84] that supported the concept that children from 2 to 10 years old would benefit the most from school re-opening. First, this age group required the presence of a caregiver at home and parents are often the only care providers available. This limits their work productivity, even when they can work at home. Moreover, children experiencing isolation and quarantine showed an increased risk of psychological disorder (such as post-traumatic stress disorder, anxiety, depression) and nutritional problems. E-learning could amplify social disparity. Finally, pre-schoolers and primary scholar did not generally use public transport. During the lockdown has long been debating on how to manage the re-opening of activities (the so-called “Phases two” and Phase three”) including schools and sports [85]. The Italian Pediatric Society has issued recommendations on the management of the extra-domestic activities of children and adolescents to facilitate the return to normality after 2 months of quarantine [86]. The principles included in the recommendations have been largely applied and can be summarized as follows: children of all age can go outdoor, respecting the social distancing and wearing a mask when indicated. The setting closest to personal residence should be choose (such as public garden, playground, park), public transports should be avoided. These recommendations have been particularly important to reduce the psychological discomfort associated with quarantine and to promote a healthier lifestyle in children and adolescence.

The SARS-CoV-2 pandemic has had deep effect on health care systems throughout affected countries. In pandemic time, the Authorities instructed the population to stay at home and to avoid clinics and hospitals as much as possible and use more tele-medicine-based practice. Consequently, Ciacchini et al [87] published in IJP an interesting report showing a 70-80% reduction in the proportion of visits at the emergency department between March 2019 and March 2020 [87] . This was confirmed by other surveys [88]. Furthermore, a group from China [89] showed that 62.86% fewer patients underwent surgery during the beginning of the pandemic (January- March 2020) compared to the same period of the previous year. The decreased number of surgeries was ascribed to confinement, to parent’s requests of deferring elective surgery and to the postponement of elective surgical activity. The reduced access to health care had also led to a decline in vaccine doses [90] administered to the children that can be detrimental for health, especially for children with special needs who are potentially at higher risk of severe illness [91]. It is of interest that a questionnaire survey in the 28 Italian pediatric scientific societies [92] found that hospital admissions, outpatient visits and specialist consultancy activities during the COVID-19 emergency were reduced with similar results both in medical and surgical areas. The main reason for reduction of pediatric care was that parents were concerned about the possibility SARS-CoV-2 transmission, and they avoided clinics and hospitals.

Another issue was the effect of COVID-19 pandemic on the work of paediatricians. Pediatric departments had to face rapid major changes to manage and prevent the spread of virus among health workers and pediatric in-patients. The most immediate and common changes [93, 94] included implementing protocols that decreased presence of healthcare workers in the hospital, promoted the use of disposable personal protective equipment, checked serum antibodies to SARS-CoV-2 [95], reduced the number of patients to preserve resources, and modified guidelines on nebulized drugs. Patients were assigned individual rooms and no visitors were allowed. Telemedicine counselling was encouraged [96–98]. However, physical examination remained crucial for diagnosis so it was important that patients could access to primary health care for assessing the need for emergency department admission [87]. IJP timely published useful recommendations of the Italian Pediatrics Society [99] on this last issue. They suggested that family pediatricians should be easily contacted by parents if they had concerns, despite the pandemic time. Pediatricians were suggested to continue providing preventive care, promoting in-person visits, immunizations, and screenings to avoid wrong diagnosis trusting on-line information [99]. It was advised to refer suspected and confirmed cases to the COVID centre to control the risk of nosocomial SARS-CoV-2 transmission. Finally, the Italian Pediatrics Society [99] stated that paediatricians should separate visits of suspected patients from all other patients with the aim of educating families in the correct use of health services and to promote hygiene strategies.

As indicated by several studies the coronavirus pandemic affected the mental health of healthcare professionals, representing a source of stress and anxiety. An American study [100] found that nearly 50% of pediatric residents had anxiety because of possible acquiring COVID-19 and spreading the infection to family members and patients. Lifestyle changes resulted in a negative effect on resident [100] and healthcare workers [101] well-being. Other studies [102, 103] showed a higher rate of depression and post-traumatic stress syndrome in health workers employed in COVID departments. Votto et al assessed the effects on the working experience, training programs and psychophysical wellbeing of pediatric residents [104]. At variance from previous studies, they did not find the development of depressive disorders psychological problems measured by Beck’s depression inventory test in residents. Residents were divided in two groups, one dedicated to Oncology, Neonatal Intensive Care, and Pediatric Inpatients Unit, the other one to Emergency Department dealing with the pandemic and no differences were found between COVID and non-COVID groups. These findings were probably due to the very low mortality rate among children during hospitalization for COVID disease. Notwithstanding, COVID-19 pandemic significantly impacted educational training, clinical practice and relationship between colleagues and required a restructuring of the daily activities. Webinars and online seminars replaced routine classroom lessons. In conclusion, it seems that appropriate measures to promote well-being are needed for selected groups of healthcare workers.

### Septic shock

Results of studies on the efficacy of dopamine versus epinephrine in children with septic shock are inconsistent [105–107]. A metanalysis by Wen et al [108] provided some insights on this matter. They compared the effects of the two drugs on shock reversal within 1 h, mortality, heart rate, mean arterial pressure and adverse events. They found that dopamine and epinephrine had similar efficacy and safety for septic shock in pediatric age. The limited number of studies did not allow to assess whether there were differences between the 2 drugs at different ages, i.e., newborns, children.

### Dacriocystitis

Early recognition and quick management for acute dacryocystitis are necessary to prevent complications due to infection spreading and achieve excellent outcomes [109]. Oral antibiotics may be sufficient in uncomplicated cases. In complicated cases or septic patients, parental antibiotics should be administered. When surgery is needed, probing and dacryocystorhinostomy for complex obstructions [110] is commonly used in adults and children. Di Cicco et al [111] showed that open dacryocystectomy was safe and effective in severe cases when imaging studies were not available.

### Thrombophlebitis

Phlebitis is a frequent complication associated with the use of peripheral intravenous catheters [112]. Septic thrombophlebitis is a potentially life-threatening condition [113] commonly due to bacteria, such as Staphylococcus aureus or coagulase-negative Staphylococci [114]. An updated review by Colomba et al [115] published during the past year, summarized the role of Candida species in thrombophlebitis. It highlighted that risk factors for the development of Candida thrombophlebitis were central venous catheters, total parenteral nutrition, and multiple antibiotic therapy, prematurity. The treatment included removal of peripheral or central intravenous catheters, antifungal therapy, and evaluation for a possible surgical excision of the affected vein. The administration of anticoagulation or thrombolytic therapy as adjunctive treatment was considered controversial.

### Microbioma. 1- Gut microbiota; 2- Probiotics

#### Gut microbiota

The intestinal microbiota is primarily composed of six microbial phyla including: Firmicutes, Bacteroidetes, Actinobacteria, Proteobacteria, Tenericutes and Fusobacteria. Notably, the dominant phyla occurring in the infant microbiota are represented by Actinobacteria (mainly Bifidobacterium) and Firmicutes [116]. In a longitudinal prospective observational study [117] the preliminary results showed that pre-pregnancy, maternal and infant factors likely impact on the composition of the infant microbiota at different levels. In particular, the relative abundance of Firmicutes increased after one month of life, if compared to meconium (T0), was related to a decrease of Proteobacteria and Fusobacteria. Furthermore, significant differences at genus and species levels were found concerning birth mode (cesarean section vs. vaginal delivery), maternal pre-pregnancy body mass index (BMI < 25 Kg/m2 vs. BMI ≥ 25 Kg/m2) and according to type of feeding mode (breastfeeding vs. formula-feeding). Turroni et al [118] presented the current state of the art regarding the infant gut microbiota and the role of key commensal microorganisms like bifidobacteria in the establishment of the first microbial communities in the human gut. Genetic and environmental factors were known to modulate the composition of the gut microbiota [119, 120], which was believed to exert key roles in modulating the immune response at both intestinal and extraintestinal sites [121].

#### Probiotics

The Italian Ministry of Health authorized the definition “probiotics” for food and food supplements under certain conditions, including a minimum number of viable cells (1 × 109 CFU) administered, a full genetic characterization of the probiotic strain and a demonstrable history of safe use in the Italian market [122]. A document from Italian Society of Pediatrics [123] summarized the current evidence on efficacy and safety of probiotics in several disorders. They concluded that well-designed trials are still needed to better understand the effect and safety in necrotizing enterocolitis, acute infectious diarrhea, allergic diseases, and functional gastrointestinal disorders. Only selected probiotics may be indicated for acute infectious diarrhea. The effects of probiotic administration for prevention/treatment of allergic diseases were still controversial. Except for breastfed children with infantile colic, the administration of probiotics cannot be recommended in children with functional gastrointestinal disorders [123, 124]. There was a growing interest in the exploitation of a next generation of probiotic bacteria [125], with the ability to modulate the gut microbiota to treat or prevent autoimmune and allergic diseases, particular types of cancers, infectious diseases [126]. Apart from the possible use of probiotic strains, such as B. bifidum PRL2010, for infants, another potential application of these probiotic bacteria included the treatment of women during pregnancy [118]. Such probiotic therapy may be crucial to colonize the mother’s gut prior to the delivery and to guarantee the settling of an appropriate bifidobacterium community that will be vertically transmitted to the newborn at birth. Nevertheless, rigorous clinical testing involving these bacteria need to be performed before meaningful probiotic claims (for humans) can be made.

### Neonatology. 1- COVID-19; 2- Global health; 3- Neonatal infection; 4- Cord blood; 5- Metabolomics; 6- Others

#### COVID-19.

At the beginning of pandemics, clinical features of newborns with COVID-19 were sparsely reported [127–129]. A review from The Study Group of Neonatal Infectious Diseases of The Italian Society of Neonatology [130], including articles published until April 07th, 2020, described clinical features of SARS-CoV-2 infection in neonates and infants up to 6 months of age. This document underlined that in the light of the limited number of cases and clinical evidence, the recommendations for management [131–133] should be constantly updated. So, it is of note that more cases were published in IJP. Olivini et al [134] described five asymptomatic or pauci-symptomatic mother with COVID-19 infection and four newborns with positive rhinopharyngeal and /or rectal swabs. Two out of four newborns showed mild respiratory and gastrointestinal symptoms. Another report [135] described two preterm newborns with respiratory distress, born to mother with COVID-19 pneumonia. Both newborn were treated with hydroxychloroquine [136]. Because of maternal anamnesis and neonatal clinical course, the authors reasonably assumed that the symptoms in both newborns were caused by prematurity or sepsis, rather than SARS-COV-2 infection [137, 138].

### Global health

Neonatal care in developing countries is an important issue. Tessema et al [139] identified pooled magnitude and determinants of postnatal care utilization in sub-Saharan Africa (SSA), using a multilevel model allowing to handle both individual and community-level characteristics. Basic health indicators included mortality, morbidity, family planning service utilization, fertility, maternal and child health. Their results confirmed the wide gap in postnatal care utilization between SSA countries [140–142]. Conflicting results have been reported by studies on risk factors for preterm birth in East Africa. It is of note that Laelago et al [143], adopting PRISMA guideline, completed the first systematic review that provided pooled data on determinants of prematurity in East Africa with the purpose of suggesting policy makers, clinicians, and program officers a design intervention to prevent prematurity. Socio economic and demographic factors, reproductive health, obstetric factors, medical condition, nutrition, and behavioural factors were considered in the whole analysis. Shorter pregnancy interval, less than 4 antenatal care visits, maternal anemia, dual infection of HIV and malaria, chorio-amnionitis, placenta praevia, untreated bacterial vaginosis quinine exposure in first trimester were correlated with preterm birth. On the other hand, dietary iron, drink home brew during pregnancy, intermittent preventive treatment with sulphadoxine-pyrimethamine were significantly associated with protection from prematurity [144–146]. Low iron levels in the first 6 months of life are associated with alteration of memory, cognitive and executive function up to adult age [147, 148]. In Northwest Ethiopia neonatal anemia identified by cord sampling was found in 25% of term newborns [149]. Socio-demographic characteristics showed a statistically significant association with maternal vegetable consumption habit. The improvement of maternal nutrition in a preventive setting and an early identification of severe neonatal anemia can be considered as main interventions plans to be pursued in an auxological and developmental perspective [150]. In SSA, low birth weight (LBW) is the major cause of neonatal mortality and morbidity [151]. In Ethiopia, several studies reported inconsistent results in the relationship between pregnancy induced hypertension and LBW. IJP has issued a systematic review and meta-analysis by Getaneh et al [152] showing that in Ethiopia about one third of babies from mother affected by pregnancy induced hypertension were low birth weight (LBW) [153], a number more than two times higher than the pooled estimate of LBW among the total population in Ethiopia. Improving the availability, accessibility and quality of maternal health care services is warranted to modify the scenario [151, 154].

### Neonatal infection

A single centre study [155] on healthcare-associated infections in a Neonatal Intensive Care Unit (NICU) comparing two study periods of 4 years (2006-10 versus 2013-17) showed that central line-associated blood stream infection and ventilator associated pneumonia (VAP) were prevalent, 67 % and 20 % respectively, over urinary tract infection and necrotizing enterocolitis, in all birth weight classes. While a decreasing trend from the lowest to the highest birth weight classes in both study periods was evident, a higher incidence of the central line-associated blood stream infection caused by Gram positive bacteria or by undetermined etiology in the first period and a lower incidence of VAP caused by Gram-negative bacteria in the second period were observed. These observations highlight the necessity of healthcare-associated infections surveillance protocol in the NICU, to plan neonatal infection interventions as well as the utility of setting a benchmarking for healthcare-associated infections on a national scale [156–158].

### Cord blood

It is to be determined whether delayed umbilical cord clamping (DCC) after elective caesarean section (ECS) is of benefit as after eutocic delivery [159, 160]. De Bernardo et al [159] compared DCC at 1 minute versus immediate cord clamping (ICC) in term infants born by ECS. No difference was reported for SpO2, heart rate, temperature both at 5 min and at 10 minutes and glycemia at 120 minutes. Main positive effect was an increased haematocrit after 72 h of life, not associated with an increased rate of phototherapy. Considering the known best iron storage between 3 and 6 months of life, less incidence of transfusion and hypotension and better neurodevelopmental trajectories, these results strongly support DCC in term infants born by ECS [161, 162].

Immediate identification of respiratory distress syndrome (RDS) and early treatment is important to reach better outcomes. So, it is important to identify early biomarkers of RDS such as blood gas analysis (BGA) to improve diagnosis [163]. In 352 full term neonates born from vaginal delivery with Apgar score > 7 at 5 minutes, arterial cord BGA results showed an association with RDS, specifically for arterial cord blood pH < 7.12, in 33.8% of infant. Additional risk factors for RDS were hypothermia and hypoglycemia. The pH level associated with admission to NICU was 7.10. Moreover, pH and lactate were the best predictive indexes for the access in NICU. The neonatologist should not be induced by a normal Apgar score at 5 minutes to exclude the possibility of a state of impending acidosis and the related risk for RDS onset [164–166].

### Metabolomics

Metabolomics continue to be a significant item of pediatric research. Bardanzellu et al [167] have reviewed clinical application of metabolomics in several clinical contexts including adult and pediatric cancer, obstetrics, perinatal asphyxia, neonatal nutrition, neonatal sepsis, and neuropsychiatric disorders [168, 169] aiming to offer updated evidence. Urinary metabolome was strictly correlated with perinatal asphyxia and concomitant multiorgan involvement, mainly liver and kidney, with high prognostic value. Metabolomic research demonstrated relations between breast feeding and metabolic programming influencing future newborns’ metabolism and was useful for early and sensible diagnosis, management, and prognosis of neonatal sepsis. Moreover, metabolomics perturbations in blood, urine, or saliva, and in cerebellum or cortex samples were associated to autism spectrum disorders. The paper provided insights that metabolomics can be useful for a more individualized nutritional and therapeutic approach in newborns and children.

### Others

There are few reports on risk factor and the effects of treatment in neonatal disseminated intravascular coagulation (DIC). In a retrospective cohort study Go et al [170] using a standardized score for neonatal DIC severity, assessed associated risk factor and effectiveness of targeted therapy. Several associated parameters and pathological conditions were considered and therapeutic approach as fresh frozen plasma (FFP), antithrombin, recombinant human soluble thrombomodulin (rTM) and a combination of them were comparatively analyzed. Small for gestational age, low Apgar score, hemangioma, hydrops, pregnancy-induced hypertension, placental abruption and mainly birth asphyxia were statistically increased in newborns with highest DIC score. The association of FFP and rTM resulted to be effective for neonatal DIC at birth. The main limit of the study was to have enrolled only one patient with neonatal sepsis. Thus, studies on rTM and FFP treatment on sepsis associated DIC in neonates are warranted [171–173].

Iatrogenic illnesses are frequent and are the result of errors in type of drug, dose, route of administration or patients in a hospital setting. The first case series about iatrogenic severe hyperglycemia (ISH), defined as glycemia > 300 mg/dl, caused by parenteral administration of glucose were reported by Bruns et al [174]. ISH [175] was found in 11 patients, ranging from premature infants to children. Most patients (73%) were treated in an intensive care unit. ISH was mainly consequence (73%) of an error in administration, 55% of infants needed intubation and 36% had poor outcome. Insulin therapy was started in 70% of patients. More prospective studies are needed to develop treatment strategies, along with measures for prevention of ISH.

Feeding intolerance in premature infants often extend NICU staying. A systematic review and meta-analysis study by Seiiedi-Biarag et al [176] underscored the benefits of massage techniques applied in the management of the preterm infant with the aim of improving intestinal motility and its effects on feeding intolerance to shorten the duration of hospitalization in NICU. Massage therapy activated the parasympathetic system resulting in an increase in intestinal motility, documented by significant reduction of gastric residual volume and vomiting in preterm infants. Furthermore, it improved weight gain and reduced gastrointestinal complications, such as necrotizing enterocolitis. This cost-effective intervention has been mainly adopted in Asian countries [177, 178]. More clinical trials with standardization of the massage method, as well as results from investigations conducted in Western countries are necessary [179]. The frequency of fetal arrhythmias is about 1% and it may have serious consequences. Yuan et al [180] have elucidated better diagnostic, treatment of sustained fetal arrhythmias potentially responsible for hydrops fetalis, cardiac dysfunction or intrauterine fetal demise. On the diagnostic side, they underlined that anatomical M-mode ultrasound, providing simultaneous two-dimensional real-time images, obtained better quality tracings of atria and ventricles than standard M-mode views. Doppler echocardiography can determine the rhythm changes between the spectra and the arrhythmic patterns, and fetal magnetocardiography allows, more than electrocardiography, real-time detection of arrhythmias. On the therapeutic side [181] different direct access to fetal circulation, not free from risks for the fetus, were well described in the manuscript, along with a detailed list of treatment of choices for fetal tachyarrhythmias [182, 183]. Overall, the paper has provided a unique useful guidance on the management of the disease.

### Neurology. 1- obsessive-compulsive disorder; 2- Listeria monocytogenes; 3- Neurological disability; 4- Screen time

#### Obsessive-compulsive disorder

All aspects of quality of life are markedly affected in individuals with obsessive-compulsive disorder (OCD) and associated severity (particularly obsessional severity) and depression severity [184]. Family accommodation (FA) was a salient phenomenon observed in pediatric OCD. It was multifaceted and can encompass a wide variety of behaviors, including modification of the family’s schedule to accommodate a subject’s obsessive-compulsive symptoms, facilitation of avoidance, engagement in compulsions and provision of OCD-related reassurance [185]. Accommodating the child’s obsessive-compulsive symptoms fed into the negative reinforcement cycle by preventing the child from facing the triggering situations [186]. Children with High Accommodation [187]. demonstrated significantly major functional impairment in global, social and role domains, and higher depressive symptoms than those with Low Accomodation. Both fathers and mothers from the High Accommodation group reported a higher level of individual psychological distress compared to mothers and fathers from the Low Accommodation group. The presence of FA should therefore be carefully investigated and considered in planning assessment and treatment of OCD in children and adolescents.

#### Listeria monocytogenes

Listeria monocytogenes is a Gram-positive, facultative intracellular bacteria that typically affects pregnant women, newborns, immunocompromised and older adults. Listeria monocytogenes [188] can be responsible for invasive infections including sepsis, brain abscesses, meningitis, meningoencephalitis and rhombencephalitis. Although rare in healthy children, beyond the neonatal period, its course can be rapid and aggressive with severe complications, such as acute hydrocephalus and high mortality [189]. Mortality rates with confirmed Listeria monocytogenes infection are approximately 15% but can be higher depending on patient status and comorbidities [190]. Hydrocephalus is rarely reported in patients with Listeria monocytogenes meningitis. So, data on the management and outcome of Listeria meningitis-related hydrocephalus in children are scarce and it is important that Brisca et al [187] have described a new case to promote appropriate care. They elucidated that in infants with meningoencephalitis due to Listeria Monocytogenes, hydrocephalus should be suspected when neurological deterioration occurs few days after initial presentation, despite adequate antibiotic treatment [191]. Therefore, a close clinical monitoring of affected children was warranted for prompt critical care interventions. Even if conservative treatment is often proposed, they underlined that invasive procedures were mandatory in patients with moderate or severe hydrocephalus and progressive clinical deterioration.

#### Neurological disability

Children with neurologically impaired (NI) have feeding difficulties and gastrointestinal symptoms, which result eventually in malnutrition and growth failure, with poor health-related quality of life [192]. Di Pasquale et al [193] highlighted that the awareness of feeding difficulties involving up to 85% of children should be increased. Furthermore, the nutritional management of children with NI should encompass thorough evaluation of nutritional status with standard anthropometrics and body composition and definition of undernutrition by physical examination and requirements. They should be calculated taking into account dietary reference intake for basal energy expenditure in typically-developing children, individualized according to motor function, muscle tone, and activity level. Prompt nutritional intervention [194] should consider enteral nutrition before the development of undernutrition. Gastrostomy should be the preferred way for prolonged enteral tube feeding. Post-pyloric feeding should be chosen in case of contraindication to gastric feeding. Enteral formula should be given [195] to evaluate the benefits of enteral tube feeding: namely long-term improvement of nutritional status, health-related quality of life and low rates of serious complications. Finally, the most frequent gastrointestinal complaints, gastroesophageal reflux and constipation should be diagnosed and treated.

#### Screen time

There is considerable evidence that higher levels of screen time are associated with a variety of health harms, with evidence strongest for adiposity, unhealthy diet, depressive symptoms, and quality of life [196]. Risks include negative effects on sleep, attention, and learning and exposure to inaccurate, inappropriate, or unsafe content and contacts and compromised privacy and confidentiality [197]. These risks are both in preschool and school-aged children [198]. Xie et al. conducted a cross-sectional study in 3.842 subjects preschool children [199] showing that male children, one-child households, rural households, single-parent households, low-income households, families with lower maternal education, families attended by grandparents or babysitters and children who slept less had more commonly a daily screen time > 60 min. Preschoolers who spent more than 60 minutes screen time tended to show a greater risk of negative effects on temperament, character and vulnerability to inattention and attention deficit and hyperactive disorder.

### Nutrition. 1- Obesity; 2- Malnutrition; 3- Vitamin B12

#### Obesity

Romanelli et al [200] summarized the risk factors for obesity [201] that can be addressed as a target of preventive interventions. The characterization of epigenetic changes and their role in childhood obesity might enable the identification of early markers of risk, therapeutic targets, and prevention strategies. Family-based programs were associated with a significant improvement in adiposity measures and cardiometabolic risk profile. The obesity prevalence can be slightly reduced through school interventions with a multicomponent approach (nutrition and physical activity). Furthermore, urbanization was one of the causes of childhood obesity as it was associated with higher consumption of junk food and sedentary behavior. Children living in rural areas had a higher risk of obesity than urban counterpart. Because of the multifactorial nature of obesity, variability in its severity, health implications, and treatment should be conducted in multiple settings. Prevention appeared the most promising tool to counteract the obesity epidemic [202]. Education toward healthy nutrition and active lifestyle constitutes the basis of every type of intervention program [203]. A multicomponent and multilevel approach involving community, school, and family might be more effective than single component programs.

#### Malnutrition

Underweight (i.e., low weight-for-age) remains one of the most common causes of morbidity and mortality among children throughout the world and is a composite measure of stunting and thinness (i.e., wasting) [204]. Short stature, also called stunting, is frequently misinterpreted as a proxy indicator for malnutrition [205]. El-Shafie et al [206] conducted a cross-sectional study in 33.150 Egyptian children aged 6–11 years old. The prevalence of underweight was 8.2% among studied children, which was higher than reports from studies in high income countries like the United Kingdom but was equal to that in low income countries. Obesity and overweight affected 21.8% of the study population (9.6 and 12.2% respectively). Short stature was found in 17% of the subjects. Main causes of short stature were familial (40.8%) and constitutional short stature (24.2%). Anemia related to nutrient deficiency [207] was diagnosed in 26% of children, while concurrent anemia and stunting were reported in 9.9% of the cases. The high prevalence of children with short stature rather than in malnourished stunted ones, showed that growth in height was not dependent only on the extent and nature of the diet and stunting was not synonymous of malnutrition. The high percentage of both undernutrition and overweight was explained by the nutritional scenarios of the developing countries due to socio-economic and demographic transition, dietary habits, lifestyle modification and increasing risks of non-communicable diseases.

#### Vitamin B12

Vitamin B12 deficiency has 3 primary etiologies: autoimmune condition, malabsorption, and dietary insufficiency [208], especially in infants born to vegan mothers. Vitamin B12 deficiency is not usually suspected by the pediatrician in healthy infants with neurological symptoms because the manifestations are nonspecific: apathy, rejection of food and loss of maturational patterns. Dubaj et al [209] reported a 7 month old girl with somnolence and developmental regression with onset approximately 2 months prior to the visit and megaloblastic anemia. It was determined that the infant's symptoms were due to vitamin B12 deficiency which was secondary to the mother's latent Addison-Biermer disease. After intramuscular vitamin B12 administration, the patient’s neurological condition improved within 1 week. However, the psychological evaluation conducted 9 months after hospitalization still indicated deficits in various areas of psychomotor development [209]. Infantile vitamin B12 deficiency may cause long lasting neurodisability even though vitamin B12 supplementation leaded to rapid resolution of severe neurological symptoms [210].

### Orthopedics. 1- COVID-19; 2- Dysplasia of the hip; 3- Congenital clubfoot

#### COVID-19

The rapid spread of the COVID-19 outbreak immediately caused the congestion of many hospitals, thus imposing the temporary interruption of all non-essential medical care. Therefore, it was important that orthopedic departments emphasize the safety of the health workers to slow the spread of the virus so that the department could still maintain its vital function [211]. Trisolino et al [212] drew up a document which provided general and specific recommendations for pediatric orthopedic surgeons, who faced the so-called “pandemic period” and the “post-peak period” with the following aims: 1) to provide essential care to pediatric patients who needed orthopedic treatments; 2) to ensure safety of children and caregivers in case of hospital admission; 3) to ensure safety of medical staff and limit the spread of the outbreak. It was necessary to cancel all deferrable outpatient appointments and follow-up visits, to reduce the circulation of users and staff within the hospital structures and to confirm the appointments for children who required post-operative care that cannot be postponed or of the cases with recent onset of symptoms or progressive exacerbation to exclude serious disease. In case of emergency surgery, it was recommended to screen the patient using rapid response SARS-CoV 2 tests (nasopharyngeal swab). If absent, or if surgery was required in less than 4–6 h, the patient should be considered COVID-19 positive, to minimize infection spread. Patients should remain in the filter area until the nasopharyngeal swab result was available.

#### Dysplasia of the hip

Dysplasia of the Hip (DDH) [213] is asymptomatic during infancy and early childhood, and, therefore, screening of otherwise healthy infants is recommended to detect the condition. Traditional methods of screening include the newborn periodic physical examination, associated with ultrasonography [214]. The goal of the treatment is to achieve and maintain a concentric reduction of the femoral head in the acetabulum to allow for continued normal development of the hip [215]. An intersociety document [216] provided recommendations for early diagnosis and treatment of DDH to reduce the possibility of hip osteoarthritis in young adults. All newborns at birth should undergo a clinical examination of the hip, which should be repeated during the first 6 months of life by the family pediatrician. Newborns presenting with a “clunk sign" should undergo the ultrasound examination of the hips before discharge from the point of birth or, in any case, within the first week of life. All infants, regardless of the presence of risk factors, should be included in a program of DDH screening which included the ultrasound examination of the hips between 4 and 6 weeks of life. Health services should identify the local DDH screening centres and care pathway, for all cases presenting with a positive dysplasia ultrasound examination (type IIb, IIc, D, III, IV image according to Graf’s classification) [217].

#### Congenital clubfoot

Clubfoot is one of the most common congenital malformation of the foot, affecting approximately 1–4 per 1000 live births [218]. If left untreated, the deformity will persist into adulthood and severely impair life quality [219]. Dibello et al. elucidated that early recognition of the deformity and immediate referral to the orthopedic specialist are the key elements for effective treatment [220]. If clubfoot was detected, a local orthopedic surgeon should be contacted to establish prompt referral to a clubfoot center, preferably within 48 h but no longer than 1 week after delivery. The diagnosis of clubfoot should be made on a clinical basis, and usually no radiological evaluation is required. Radiological evaluation could be proposed in selected cases (for example, when a weak response to treatment or severe relapses occur). Non-surgical treatment was recommended because of the higher risk of complications of surgical than non-surgical treatment. Non-surgical treatment of clubfoot has resulted in excellent success rates and overall outcomes [221]. In severe forms, parents should be alerted about the increased risk of relapse.

### Respiratory tract illnesses. 1- Cystic fibrosis; 2- Community acquired pneumonia; 3- COVID-19; 4- Mechanical ventilation

### Cystic fibrosis

Cystic fibrosis (CF) is an autosomal recessive disorder due to mutations in CFTR gene leading to abnormality of chloride channels in mucus and sweat producing cells. Respiratory system and gastrointestinal are primarily involved, but eventually multiple organs are affected leading to life threatening complications. Management requires drug therapy, extensive physiotherapy, and nutritional support. At present over 100,000 people suffer from this disease worldwide. Studies measuring psychological distress in individuals with cystic fibrosis (CF) and caregivers have found high rates of both depression and anxiety [222]. Psychological symptoms in both individuals with CF and parent caregivers have been associated with decreased lung function, lower body mass index, worse adherence, worse health-related quality of life, more frequent hospitalizations, and increased healthcare costs [223]. Continisio et al [224] showed a significant presence of stress in females (60.23%), subjects married (84.62%), unemployed subjects (69.23%) and "middle School" education levels (61.54%). Authors also highlighted a positive correlation between clinical severity and total stress, i.e., an increase of abnormal stress was found in parents who were concerned because of severe course of the disease in their kids. A significant correlation was found between stress levels and number of siblings, i.e., the presence of more children in the same family was associated with high parental stress levels. A comprehensive model of management with a regularly provided psychological support appears to be a key factor for coping and achieving a strong therapeutic alliance between healthcare professionals and patients with CF. For this reason, the data presented should be interpreted with caution, although they pave the way to future research on CF patients’ psychometric properties. Despite such limitations, the study has demonstrated that the clinical management of CF children should include a psychological support to parents since parenting stress does play a fundamental role in the effectiveness and efficacy of treatments and affects patients’ quality of life.

Respiratory viruses are known to cause pulmonary exacerbations and substantial morbidity in patients with CF [225]. Respiratory viruses are detected approximately in 28 to 48% of CF patients; hence, viruses may be important triggers of airways exacerbation [225]. In consideration of the severity of the COVID-19 infection and the high transmissibility of the virus, advice was addressed in many countries at the outset of the global pandemic to CF patients and their families to reduce the chances of contracting the virus [226]. However, SARS-CoV-2 infection rate was lower than in patients with CF that in the general population [227]. A milder form of COVID-19 might be expected because disruption of IL-6 signaling in CF lungs occurs through elevated serine protease release and subsequent cleavage of both membrane-bound and soluble IL-6 receptors. Chronic pharmacological therapies, including azithromycin (immunomodulator) and DNase (mucolytic), administered to 56 and 51% of CF patients, respectively, might also contribute to the protection. CF patients generally undergo vitamin D supplementation, which may have a protective effect against COVID-19. Taken all together, these data strengthen the hypothesis that CF may constitute an advantage in COVID-19, encouraging the scientific community to look for the possible reasons behind this unexpected scenario.

#### Community acquired pneumonia

Differentiating bacterial from viral pneumonia based on clinical characteristics is challenging as the clinical signs and symptoms overlap [228]. Rise or drop in the concentration of a single marker is not accurate enough for predicting viral/bacterial CAP [229]. This is because there is overlapping to a varying extent depending on the marker cut-off values, detection methods, analyses, the desired specificity, and sensitivity. Furthermore, the presence of mixed infection makes almost all markers suboptimal to be used universally. Another approach is to make use of more than one marker and combine with clinical signs and symptoms. This may not be cost-effective in many clinical settings; nevertheless, in many studies, marker combination greatly improved the predictive power [229]. There are several promising markers to distinguish between viral infections and bacterial ones. Myxoma resistance protein (MxA1) is increased in viral pneumonia but not in bacterial pneumonia. It has been hypothesized that if a bacterial infection is diagnosed and high levels of MxA1 are detected, this is an indication that bacterial organism preceded a viral cause [230] since MxA1 is elevated for approximately ten days after a viral infection. High mobility group box one protein (HMGB1) is elevated during CAP, sepsis and viral-bacterial co-infections, especially bacterial and influenza virus co-infection. Lipocalin 2 when combined with clinical features is 82% sensitive and 91% specific for bacterial CAP [231]. It is very challenging to accurately predict bacterial or viral pneumonia based on clinical features, or on radiological or laboratory results. New and advanced technologies capable of sensitively and specifically discriminating viral, bacterial, and viral-bacterial coinfections are needed.

#### COVID-19

In recent years, point-of-care ultrasound (POCUS) is being increasingly used in pediatrics, as it is rapid, portable, repeatable, and non-ionizing. Specific patterns of lung ultrasound (LUS) have shown to be useful in the differential diagnosis of pneumonia or acute bronchiolitis, with potential for prognosis [232, 233]. LUS should be used in children with persistent cough in whom COVID-19 is suspected because it can highlight undiagnosed interstitial lung lesions and reinforce the diagnostic suspicion of COVID-19 [234]. The routine utilization of LUS in the evaluation of children with suspected or confirmed COVID-19, when performed by clinicians with documented experience in LUS, seems useful in diagnosing and monitoring pediatric COVID-19 pneumonia, reducing unnecessary radiation/sedation in children and exposure of health care workers to severe acute respiratory syndrome due to Coronavirus [235].

Pulmonary function tests (PFTs) are aerosol-producing procedures which require maneuvers such as deep and force expiration without a mask. This causes a wide spread of droplets from subjects infected with SARS-CoV2. The Italian Pediatric Respiratory Society has developed recommendations for pulmonary function testing laboratories during the COVID-19 pandemic [236]. A specific regulatory protocol has been signed on March 14th, 2020, by unions and companies in agreement with the Italian government to protect the health and safety of workers from possible infection with SARS-CoV-2 and ensure the safety of the work environment [237]. Because of the high contamination risk of COVID-19 among patients and the pulmonary function laboratory staff, spirometry should be performed during the pandemic period in children with chronic lung disease and only if the test will guide the management. During the pandemic phase and post peak phase, clinicians should restrict referrals for spirometry to those patients who require it urgently or when it is essential for the diagnosis. PFTs should be avoided in febrile patients (body temperature < 37.5 °C) and any sign/symptom of respiratory disease (e.g., cough, shortness of breath). Measures for COVID-19 prevention and control should be adopted. Disposable materials (filters, mouthpieces, nose clips, flow sensors) to be discarded after a single use, cleaning and disinfection procedures for environment and equipment are recommended. Immunocompromised patients should be scheduled for testing at the start of the day, before other patients, whereas infected patients should be scheduled for testing at the end of the day to prevent cross-infection. Patients should be isolated in a separate area for testing to minimize contacts with potentially infectious patients in addition to standard measures for preventing and controlling cross-infection acquired from the PFT laboratory. The use of nebulizers should be avoided to decrease the risk of disseminating COVID-19 to other patients and to physicians, nurses, and other personnel in the laboratory, unless an airborne infection isolation room is available [238].

#### Mechanical ventilation

Noninvasive ventilation (NIV) refers to the use of techniques to deliver artificial respiration to the lungs without the need for endotracheal intubation. NIV has proven beneficial in comparison to invasive mechanical ventilation (IMV), and it has become the first line of treatment for paediatric respiratory distress in many countries [239, 240]. Theoretically, IMV protects the airways from aspiration pneumonia and may ease the clearance of bronchial secretions [241]. Pavone et al [242] retrospectively 432 children were included in the study with Long Term Ventilation (LTV) defined as IMV or NIV, performed daily, at least 6 h/day, for a period of at least 3 months. Three hundred and fifteen cases (72.9%) received NIV; 117 cases (27.1%) were treated by IMV. The most frequent underlying condition was neuromuscular diseases (132; 30.6%), followed by upper respiratory airway diseases (107; 24.8%), central nervous system diseases (98; 22.7%). IMV was likely associated with younger age at starting ventilatory support, more hours on ventilation, more severe illness, and home health care. Overtime 39 children improved (9%), 11 children on NIV (3.5%) received tracheostomy; 62 children died (14.3%); and 74 children (17.1%) were lost to follow-up (17.8% on NIV, 15.4% on IMV. The results confirm the need for high-quality health care programs able to promote well-being of children on long-term ventilation.

### Rheumatology. 1- Juvenile idiopathic arthritis

Harlequin ichthyosis is a rare autosomal recessive ichthiosys with an incidence of less than 1/300.000 births [243] that is associated with perinatal morbidity and low neonatal survival rate. Early treatment with oral retinoids is recommended for increasing survival. A Th-17/IL-23 polarization has been found in some ichthyoses [244]. Th17 cells play also a pathogenetic role in juvenile idiopathic arthritis [245]. It is therefore of note that Auriti et al [246] described 2 patients with Harlequin ichthyosis who developed juvenile idiopathic arthritis [247] that has an incidence of 15–150/100.000 in the first 2 years of life. The observation of an association between these rare diseases [245] highlights that clinicians should be aware of onset of juvenile idiopathic arthritis in patients with Harlequin ichthyosis and promptly identify it. Furthermore, additional understandings on the pathophysiology of the relationship between the two diseases may elucidated whether novel treatments such as IL-17 targeting drugs, may be of benefit.

## Conclusion

Last year, intensive efforts have been made to understand which modifiable factors can favour the onset of pediatric diseases. Great advances have been made in understanding the pathomechanisms of diseases. These can be useful for new beneficial approaches. Several studies have offered new techniques which will be of great assistance for diagnosis. Innovative treatments have allowed to improve disease control. We expect that this review would be helpful to improve patient care.

## Data Availability

Data sharing is not applicable to this article as no datasets were generated or analysed during the current study.

## Abbreviations

ADPKD: Autosomal Dominant Polycystic Kidney Disease
BGA: Blood Gas Analysis
BMI: Body Mass Index
CDC: Centers for Disease Control and Prevention
COVID-19: Coronavirus Disease
CF: Cystic Fibrosis
DCC: Delayed Cord Clamping
DKA: Diabetic Ketoacidosis
DIC: Disseminated Intravascular coagulation
DDH: Dysplasia of the Hip
ECS: Elective Caesarean Section
FMF: Familial Mediterranean Fever
FFP: Fresh Frozen Plasma
ISH: Iatrogenic Severe Hyperglycemia
ICC: Immediate Cord Clamping
T1D: Insulin Dependent Diabetes Type 1
ICU: Intensive Care Unit
IMV: Invasive Mechanical Ventilation
IJP: Italian Journal of Pediatrics
LBW: Low Birth Weight
LUS: Lung Ultrasound
DIC: Neonatal Disseminated Intravascular Coagulation
NI: Neurologically Impaired
NIV: Non-invasive Ventilation
OCD: Obsessive-Compulsive Disorder
POCUS: Point-Of-Care Ultrasound
PKDs: Polycystic Kidney Diseases
PFTs: Pulmonary Function Tests
PICCO: Pulse Index Contour Cardiac Output
rTM: Recombinant Human Soluble Thrombomodulin
RTH: Resistance To Thyroid Hormone
RDS: Respiratory Distress Syndrome
SARS-CoV-2: Severe Acute Respiratory Syndrome Coronavirus-2
TRH: Thyrotropin-Releasing Hormone
T1D: Type-1 Diabetes
VAP: Ventilator Associated Pneumonia
